# Exploring genetic variability of *Giardia duodenalis* and *Enterocytozoon bieneusi* in raw vegetables and fruits: implications for food safety and public health in Mozambique

**DOI:** 10.3389/fmicb.2023.1223151

**Published:** 2023-08-04

**Authors:** Cátia Salamandane, Maria Luísa Lobo, Sónia Afonso, Lihua Xiao, Olga Matos

**Affiliations:** ^1^Group of Opportunistic Protozoa/HIV and Other Protozoa, Global Health and Tropical Medicine, Medical Parasitology Unit, Instituto de Higiene e Medicina Tropical, Universidade Nova de Lisboa, Lisboa, Portugal; ^2^Nova School of Business and Economics, Universidade Nova de Lisboa, Carcavelos, Portugal; ^3^Faculdade de Ciências de Saúde, Universidade Lúrio, Nampula, Mozambique; ^4^Parasitology Department of Veterinary Faculty, Universidade Eduardo Mondlane, Maputo, Mozambique; ^5^College of Veterinary Medicine, South China Agricultural University, Guangzhou, Guangdong, China; ^6^Environmental Health Institute, Faculdade de Medicina da Universidade de Lisboa, Lisboa, Portugal

**Keywords:** intestinal protozoa, microsporidia, raw horticultural products, foodborne diseases, zoonotic transmission, Maputo, One Health

## Abstract

*Giardia duodenalis* and *Enterocytozoon bieneusi* are etiological agents of enteric diseases characterized by diarrhea that can progress to chronicity in humans, especially in children and in immunocompromised patients. This study aims to assess the genetic pattern of *G. duodenalis* and *E. bieneusi* detected in vegetables and fruits commercialized in Maputo markets, Mozambique and determine their public health importance. Eight study points were sampled: a farmer zone, a wholesale, four retail markets, and two supermarkets in Maputo city, where eight types of horticultural products were purchased. Using nested-PCR methods, 2.8% (9/321) and 1.3% (4/321) of samples monitored were positive for *G. duodenalis* and *E. bieneusi*, respectively. Based on the analysis of the β-giardin and ITS rRNA sequences of *G. duodenalis* and *E. bieneusi* detected, respectively, four different sequences of *G. duodenalis* (three novel sequences: BgMZ1, BgMZ2, and BgMZ3, and one known sequence) all from assemblage B and three genotypes of *E. bieneusi* (two novel sequences: EbMZ4 and EbMZ5, and one known sequence: KIN-1) from group 1. These microorganisms were found and characterized for the first time in horticultural products in Maputo markets. All identified *G. duodenalis* and *E. bieneusi* display high genetic similarity within their β-giardin and ITS rRNA sequences, respectively, having been clustered into assemblages and genotypes with high zoonotic transmission potential. Our study may represent a relevant step in the understanding of these intestinal pathogens in association with fresh vegetables and fruits for human consumption, for a better and broader “One Health” approach.

## 1. Introduction

*Giardia duodenalis* and *Enterocytozoon bieneusi* are etiological agents of acute and chronic disorders including diarrhea, malabsorption, and severe debilitation illnesses in humans and animals, particularly in children and immunocompromised patients (Lobo et al., 2014; Xiao and Feng, 2017; Stentiford et al., 2019; Junaidi et al., 2020; Rafiei et al., 2020). Human infections by the pathogens *G. duodenalis* and *E. bieneusi* may be acquired through direct contact with infected persons (person-to-person transmission) or animals (zoonotic transmission) or even ingestion of contaminated water (waterborne transmission; Baldursson and Karanis, 2011; Efstratiou et al., 2017a; Ahmed et al., 2018) and food (foodborne transmission; Li et al., 2012; Matos et al., 2012; Stentiford et al., 2016; Squire and Ryan, 2017; Rodrigues et al., 2020). As *G. duodenalis* cysts and *E. bieneusi* spores are robust and resistant to environmental conditions, some waterborne and foodborne outbreaks mostly for human giardiasis have been reported (Thompson et al., 2008; Ahmed et al., 2018; Javanmard et al., 2020; Bahramian et al., 2021; Hassan et al., 2021). Relatively less is known about the occurrence and molecular epidemiology of these two pathogenic species in water and food in Africa.

*Giardia duodenalis* has eight genetic assemblages (A-H): A, B, C, D, E, and F can infect humans (mostly A and B) and other animals, and assemblages C to H have a narrower host range (Yaoyu and Xiao, 2011; Cai et al., 2021; Dixon, 2021). *Enterocytozoon bieneusi* is mentioned as the most prevalent among the 17 human-infecting microsporidia species (Lobo et al., 2012; Matos et al., 2012; Stentiford et al., 2019). The genetic variability of this pathogenic species is based on the analysis of single-nucleotide polymorphisms (SNPs) in the internal transcribed spacer (ITS) region of the ribosomal RNA (rRNA; Santin and Fayer, 2009; Lobo et al., 2014; Li et al., 2019b). To date, over 600 genotypes of *E. bieneusi* have been identified (Cunha et al., 2017; Chen et al., 2019; Zhang et al., 2020; Cao et al., 2021), clustered in 11 phylogenetic groups (Matos et al., 2012; Xiao and Feng, 2017; Cacciò et al., 2018; Cao et al., 2021). Within Group 1, genotypes D, Ebp C, and Type IV are those most frequently found not only in humans but also in domestic and wild animals worldwide, suggesting a low level of host specificity and the potential for zoonotic or cross-species transmission. Also, Group 1 genotypes, such as Peru 6, Peru 8, and Peru 11, have been regularly identified in both humans and animals. Group 2 genotypes were once considered to be adapted to ruminants but have subsequently been found in other animals and humans. Group 3 to Group 11 genotypes seem to have a clear and strong host specificity although few information is available for those groups (Santin and Fayer, 2009; Wang et al., 2016; Li et al., 2019a,b, 2020b; Wu et al., 2020a). However, the lack of information on risk factors or epidemiology associated with its transmission limits the full understanding and implications of *E. bieneusi*'s zoonotic potential.

Several studies (Lobo et al., 2014; Li et al., 2020b; Salamandane et al., 2021b; Mozer et al., 2022) for the molecular characterization of *G. duodenalis* and *E. bieneusi* have been carried out, but in Africa, studies on *G. duodenalis* are more abundant than studies on *E. bieneusi*. More precisely, there are studies done mainly in humans and livestock (Akinbo et al., 2012; Samra et al., 2012; Lobo et al., 2014; Laatamna et al., 2015; Wegayehu et al., 2020). In Mozambique, the first study on *E. bieneusi* genotyping was recently carried out in young children, in which a novel subtype, HhMzEb1, was reported (Muadica et al., 2020).

In southern Africa, some of the few studies on these two pathogenic microorganisms were also performed in water (Dalu et al., 2011; Mtapuri-Zinyowera et al., 2014; Archer et al., 2020; Siwila et al., 2020). Globally, the occurrence of protozoan parasitic contamination in vegetables and fruits ranges from 1.9 to 9.3% (Li et al., 2019a). Thus, this study aims to carry out the genetic characterization of *G. duodenalis* and *E. bieneusi* detected in vegetables and fruits commercialized in Maputo markets, Mozambique and determine their public health importance.

## 2. Materials and methods

### 2.1. Sampling

This study was carried out in the rainy (February to March) and in the dry seasons (August to October) of 2019, in eight points of study, and according to the markets' typology, namely, a producer zone (Infulene Valley), one wholesale market (Zimpeto), four retail markets (Xipamanine, Benfica, Central da Baixa, and Fajardo), and two supermarkets (LMC and LSP) in Maputo city. In each selected study point, we randomly purchased three specimens of each of the eight types of raw and fresh horticultural products, namely, coriander (*Coriandrum sativum*), parsley (*Petroselinum crispum*), Portuguese cabbage (*Brassica Oleracea costata*), pointed white cabbage (*Brassica oleracea capitata*), carrot (*Daucus carota*), tomato (*Solanum lycopersicum*), green pepper (*Capsicum annuum*), and lettuce (*Lactuca sativa cea*). The products were placed in individual plastic bags, encoded with a unique number and the date of collection, and transported in a container at 4°C. Several detaching and concentrating *G. duodenalis* cysts and *E. bieneusi* spores protocols reported in the literature were reviewed (Monge and Chinchilla, 1996; Cook et al., 2007; Mozer et al., 2022), and the more consensual approach was selected for this study, taking into account the distinct size and food matrices of the products studied and the field work conditions.

A total of 321 samples collected (153 in the rainy season and 168 in the dry season) were transported to the laboratory for processing. At the laboratory, we first weighed the fruits and vegetables according to their sizes: for the large ones, we weighed 150–250 g (lettuce, pointed white cabbage, and Portuguese cabbage), and for the small ones, we weighed 80–100 g (coriander, parsley, carrot, tomato, and green pepper). Then, they were washed in 200 ml and 50 ml of a solution of NaCl (0.85%) in distillate water, for large and small fruits/vegetables, respectively, in a plastic bag. They were shaken vigorously on an orbital shaker (SK-300 Lab Companion, Woburn, Massachusetts, USA) at 150 rpm for 20 min and brined overnight. The next morning, the solution was collected in 50 ml Falcon tubes and centrifuged at 8,000 × *g* for 15 min. Finally, 5–8 ml of the concentrated vegetable extracts was saved for DNA extraction and direct microscopy analysis, and the remainder was used to make smears for staining (Salamandane et al., 2021b). The samples were stored in a cooler at 4°C for DNA extraction.

### 2.2. Molecular detection of *Giardia duodenalis* and *Enterocytozoon bieneusi* using nested-PCR methods

Genomic DNA was extracted from concentrated vegetable/fruit extracts for the molecular detection of *G. duodenalis* and *E. bieneusi*, using the QIAamp Fast DNA Stool Mini Kit according to the manufacturer's instructions. The extracted DNA (100 μl/sample) was kept at −20°C.

A nested-PCR analysis (Cacciò et al., 2002; Lalle et al., 2005) targeting the β-giardin (*bg*) gene was used to detect *G. duodenalis*. The primary reactions were carried out using the primers GiaFW1 (5′-AAGCCCGACGACCTCACCCGCAGTGC-3′) and GiaRV1 (5′-GAGGCCGCCCTGGATCTTCGAGAC GAC-3′) for the 753-bp fragment amplification. For the secondary reactions, the primers GiaFW2 (5′-GAACGAACGAGATCGAGGTCCG-3′) and GiaRV2 (5′-CTCGACGAGCTTCGTGTT-3′) were used to amplify the 511-bp fragment.

The detection of *E. bieneusi* was performed by nested-PCR analysis targeting the entire internal transcribed spacer (ITS) region of the rRNA gene, and its genotyping was conducted by sequence analysis of amplified fragments of the ITS rRNA gene (Lobo et al., 2012). Primary and secondary reactions were carried out using the primers MLLF1 (5′-CGCCCGTCACTATTTCAGAT-3′) and MLLR1 (5′-GCTTAAGTCCAGGGAGTATCCA-3′) to amplify a 514-bp fragment, and MLLF2 (5′-AGTCGTAACAAGGTTTCAGTTGG-3′) and MLLR2 (5′-GGACTTTTCGCATTCTTTCG−3′) to amplify a 386-bp fragment, respectively.

Successively, the PCR products were loaded on 1.5% agarose gel stained with ethidium bromide and subjected to electrophoresis and finally visualized under UV light on a proper device.

### 2.3. *Giardia duodenalis* and *Enterocytozoon bieneusi* sequencing and data analysis

The products of the expected size were purified using a PureLink™ Quick Gel Extraction and PCR Purification Combo kit (Invitrogene, Carlsbad, Califórnia, EUA), as described on the manufacturer's instructions, and were submitted to Sanger sequencing in both directions on an ABI3100 automated sequencer (Applied Biosystem, Foster City, CA) at STABVIDA Company (Lisbon, Portugal). Three PCR products of each positive sample obtained from different PCRs were submitted to DNA sequencing. The authors analyzed all three sets of sequencing chromatograms obtained for each of the positive samples. Next, the individual sequences were subsequently aligned against reference sequences from the GenBank database using the BLASTN (www.ncbi.nlm.nih.gov) and ClustalX (ftp://ftp-igbmc.u-strasbg.fr/pub/ClustalX/) programs to determine *G. duodenalis* and *E. bieneusi* genetic variability.

Representative nucleotide sequences of *bg* and ITS genes of *G. duodenalis* and *E. bieneusi* are available in the NCBI GenBank database under the accession numbers ON924995, ON924996, OP066417-OP066422, and OP076835-OP076838, respectively.

### 2.4. *Giardia duodenalis* and *Enterocytozoon bieneusi* phylogenetic analysis

Phylogenetic analysis based on the β-giardin and ITS rRNA sequences dataset of *G. duodenalis* and *E. bieneusi*, respectively, was performed using the maximum likelihood method based on the general time reversible model conducted in MEGA 7.

## 3. Results

After nested-PCR analysis, *G. duodenalis* and *E. bieneusi* were detected in 2.8% (9/321) and 1.3% (4/321), respectively, of the samples monitored. According to the distinct seasons, *G. duodenalis* was found in three (1.96%) samples (lettuce, green pepper, and tomato) in the rainy season and in six (3.6%) samples (Portuguese cabbage, lettuce, parsley/2, and pointed white cabbage/2) in the dry season. *Enterocytozon bieneusi* DNA was detected in one (0.7%) sample (pointed white cabbage) in the rainy season and in three (1.8%) samples (lettuce, tomato, and coriander) in the dry season, among four different markets. *Giardia duodenalis* was detected in products from four markets and one supermarket, and *E. bieneusi* was found in products from three markets and in products from the same supermarket as *Giardia*. The distribution of *G. duodenalis* assemblages and *E. bieneusi* genotypes identified in the horticultural products among the markets monitored, during dry and rainy seasons, in Maputo City is summarized in Table 1.

**Table 1:** Genetic characterization of *Giardia duodenalis* and *Enterocytozoon bieneusi* by nested-PCR and DNA sequencing analysis according to horticultural product, market, and seasons monitored in Maputo city.

**Sample code**	**Market**	**Season**	**Horticultural product**	**Species**	**Assemblage or genotype**
BGM Z3	Zimpeto	Rainy	Lettuce	*G. duodenalis*	B^a^
BGM Z7	Zimpeto	Rainy	Green pepper	*G. duodenalis*	B^b^
BGMZ 25	Benfica	Rainy	Tomato	*G. duodenalis*	B^a^
BGMZ 54	Zimpeto	Dry	Portuguese cabbage	*G. duodenalis*	B^c^
BGMZ 60	Xipamanine	Dry	Lettuce	*G. duodenalis*	B^c^
BGMZ 64	Xipamanine	Dry	Parsley	*G. duodenalis*	B^c^
BGMZ 69	LMC	Dry	Pointed white cabbage	*G. duodenalis*	B
BGMZ 71	LMC	Dry	Parsley	*G. duodenalis*	B
BGMZ 90	Fajardo	Dry	Pointed white cabbage	*G. duodenalis*	B
EBMZ 30	Central da baixa	Rainy	Pointed white cabbage	*E. bieneusi*	EbMZ4^d^
EBMZ 73	LMC	Dry	Lettuce	*E. bieneusi*	EbMZ5^e^
EBMZ 86	Benfica	Dry	Tomato	*E. bieneusi*	KIN-1
EBMZ 95	Fajardo	Dry	Coriander	*E. bieneusi*	KIN-1

a BgMZ1.

b BgMZ2.

c BgMZ3: new *G. duodenalis* bg sequences identified in this study.

d, e New *E. bieneusi* genotypes identified in this study.

DNA Sanger sequencing was successfully achieved for all nine and four specimens diagnosed as positive for *G. duodenalis* and *E. bieneusi*, respectively, using nested-PCR methods. The authors analyzed the three sets of sequencing chromatograms obtained for each positive sample, which were found to be consistent, indicating the reliability of the identified new sequences. No instances of mixed infection were identified.

Sequencing and alignment of our nine nucleotide sequences with others of known identity deposited in the GenBank database showed the presence of four different *G. duodenalis bg* sequences all from assemblage B in the samples studied. One of them was a known *bg* sequence, and three were novel *bg* sequences. Of the nine sequences obtained, three (BGMZ69, BGMZ71, and BGMZ90 samples) had 100% similarity with the *G. duodenalis* nucleotide sequence MK033096, one (BGMZ7) constitutes a novel sequence (BgMZ2) and differs from the previous three by a single nucleotide at 356 (G→ A) displaying 99.81% homology with sequence MK033096. The remaining five sequences generated in this study are also new *G. duodenalis bg* sequences and have 99% homology with *G. duodenalis* nucleotide sequences previously reported: three (BGMZ54, BGMZ60, and BGMZ64; BgMZ3) had one substitution to isolate KY320578 at 456 (C→ T), and two (BGMZ3, BGMZ25; BgMZ1) had three substitutions to isolate MG736246 at 99 (T→ C), at 136 (A→ G), and at 356 (G→ A).

The four *E. bieneusi* ITS sequences characterized in the four positive samples obtained in this study revealed high genetic diversity: two (EBMZ86 and EBMZ95) sequences with the genotype KIN-1 (e.g., identified in humans and livestock, such as cattle, pig, goat, horse, wild animals, and non-human primates), one (EBMZ30; EbMZ4 genotype) of the sequences characterized is a novel genotype displaying 99.71% homology with KIN-1 and Peru8 genotypes, with one substitution at 139 (T→ C) and one substitution at 159 (C→ T), respectively. The remaining novel one (EBMZ73; EbMZ5 genotype) had 99.44% similarity with KIN-1 and had two substitutions at 124 (G→ A) and at 182 (G→ A).

To obtain information on the β-giardin and ITS rRNA sequences in this study and the respective reference sequences published in previous studies, two phylogenetic trees were constructed.

**Figure 1:**
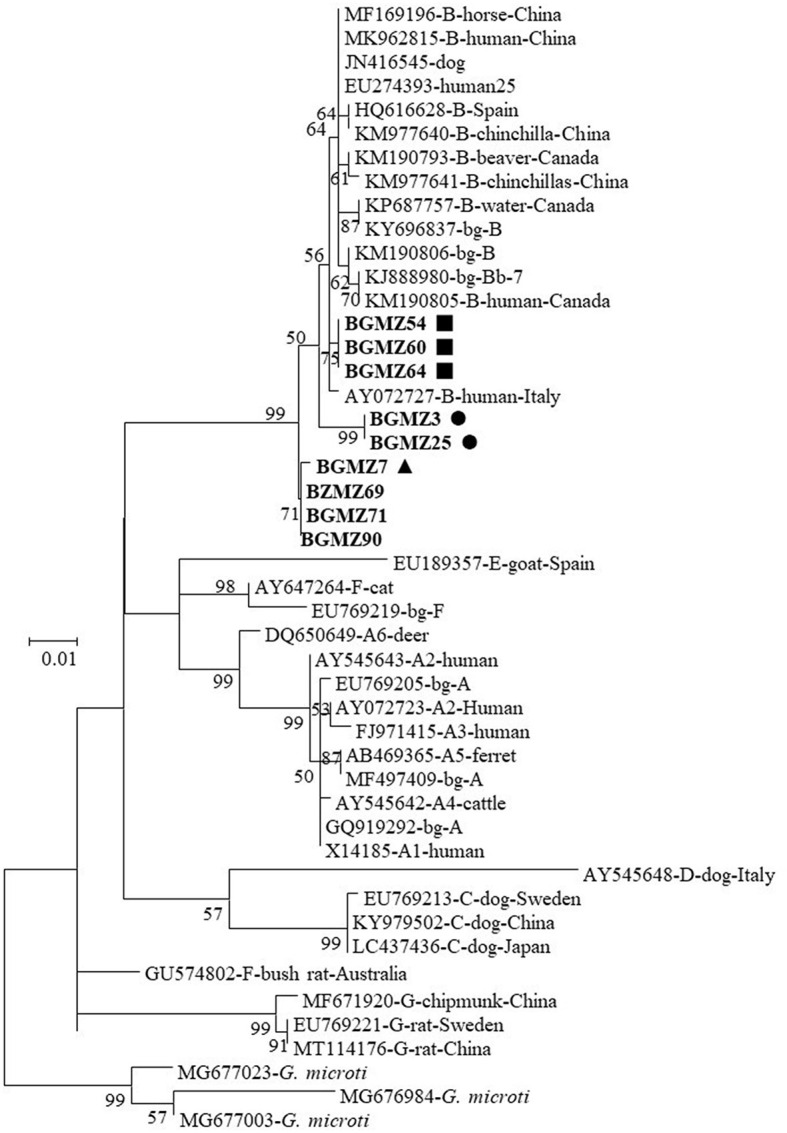
Phylogenetic relationship between *Giardia duodenalis* characterized in this study and other sequences in GenBank by analysis of β-giardin sequences. The evolutionary history was inferred by using the maximum likelihood method based on the general time reversible model. The tree with the highest log likelihood (−1,638.69) is shown. The percentage of trees in which the associated taxa clustered together is shown next to the branches. Initial tree(s) for the heuristic search were obtained automatically by applying Neighbor-Join and BioNJ algorithms to a matrix of pairwise distances estimated using the maximum composite likelihood (MCL) approach and then selecting the topology with superior log likelihood value. A discrete gamma distribution was used to model evolutionary rate differences among sites [five categories (+*G*, parameter = 0.2391)]. The rate variation model allowed for some sites to be evolutionarily invariable [[+*I*], 58.88% sites]. The tree is drawn to scale, with branch lengths measured in the number of substitutions per site. The analysis involved 48 nucleotide sequences. Codon positions included were 1st+2nd+3rd+Non-coding. All positions containing gaps and missing data were eliminated. There were a total of 472 positions in the final dataset. Evolutionary analyses were conducted in MEGA7. The *G. duodenalis* β-giardin sequences from this study are in boldface. The circles (BgMZ1), triangles (BgMZ2), and squares (BgMZ3) filled in black indicate new *bg* sequences identified in this study.

Phylogenetic analysis revealed that the nine *G. duodenalis* sequences from this study were clustered within assemblage B and formed three genetic clusters comprising species mostly infecting humans and several other domestic and wild mammals (Figure 1).

**Figure 2:**
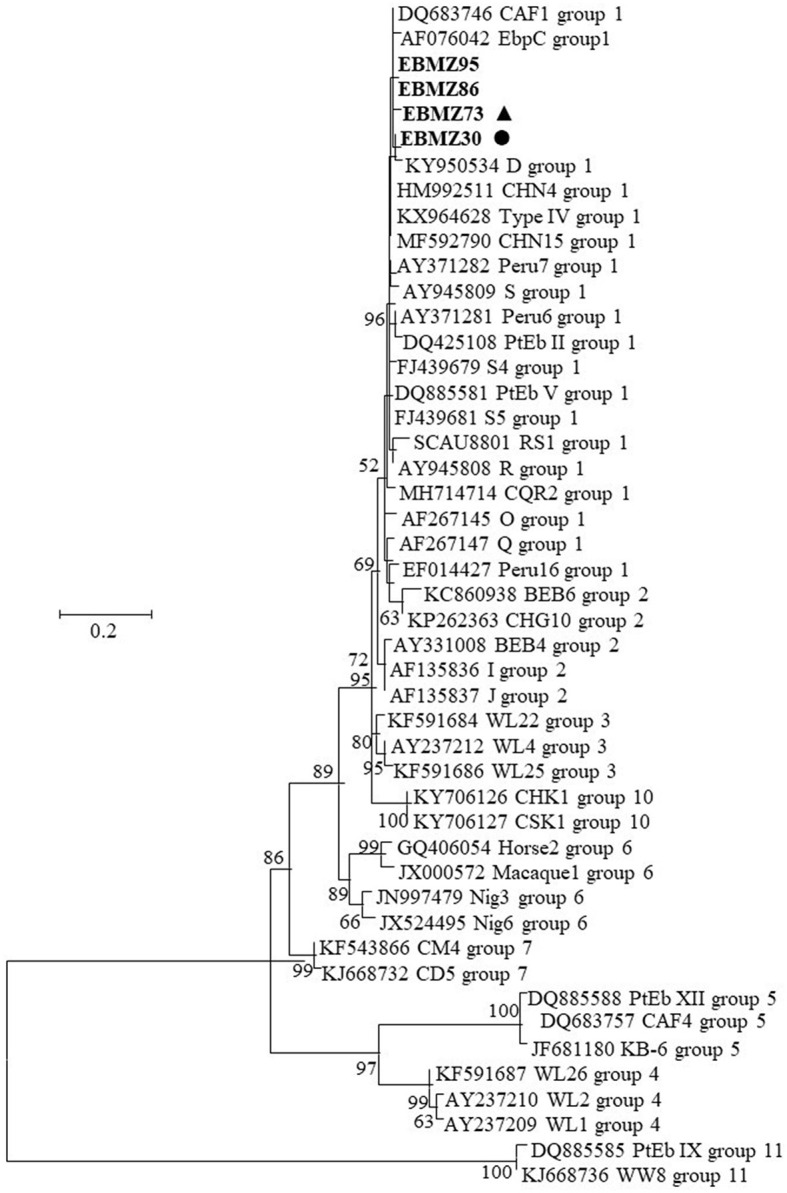
Phylogenetic relationship between *Enterocytozoon bieneusi* characterized in this study and other sequences in GenBank by analysis of ITS rRNA sequences. The evolutionary history was inferred by using the maximum likelihood method based on the general time reversible model. The tree with the highest log likelihood (−1,808.47) is shown. The percentage of trees in which the associated taxa clustered together is shown next to the branches. Initial tree(s) for the heuristic search were obtained automatically by applying Neighbor-Join and BioNJ algorithms to a matrix of pairwise distances estimated using the maximum composite likelihood (MCL) approach and then selecting the topology with superior log likelihood value. A discrete gamma distribution was used to model evolutionary rate differences among sites [five categories (+*G*, parameter = 1.4711)]. The rate variation model allowed for some sites to be evolutionarily invariable [[+*I*], 4.58% sites]. The tree is drawn to scale, with branch lengths measured in the number of substitutions per site. The analysis involved 47 nucleotide sequences. All positions containing gaps and missing data were eliminated. There was a total of 215 positions in the final dataset. Evolutionary analyses were conducted in MEGA7. The *E. bieneusi* sequences from this study are in boldface. The circle (EbMZ4) and triangle (EbMZ5) filled in black indicate the new genotypes identified in this study.

The two novel and the two known *E. bieneusi* genotypes detected in this research were all clustered into zoonotic group 1 (Figure 2).

## 4. Discussion

Several pathogens are responsible for causing human diarrheal diseases, among which *G. duodenalis* and the microsporidian *E. bieneusi* are important contributors worldwide. Both species can be transmitted by ingestion of contaminated food and water. Contact and ingestion of contaminated water are considered important risk factors related to both *G. duodenalis* and *E. bieneusi* infection in epidemiological studies (Santin and Fayer, 2009; Li et al., 2012; Squire and Ryan, 2017; Stentiford et al., 2019; Dixon, 2021). Either *G. duodenalis* cysts or microsporidian spores are robust and can be highly resistant to external environmental conditions and to several physical and chemical disinfection methods used in drinking water plants, swimming pools, and irrigation systems. Moreover, as the other human-infecting microsporidia, *E. bieneusi* spores are microscopic in size and have low specific gravity, which facilitates their easy dissemination (Matos et al., 2012; Stentiford et al., 2019; Dixon, 2021).

Fresh vegetables and fruits, despite the health benefits of their human consumption, can be an important source of some foodborne pathogenic microorganisms, including *G. duodenalis* and *E. bieneusi* (Rafael et al., 2017; Utaaker et al., 2017; Ferreira et al., 2018; Li et al., 2019a, 2020a; Archer et al., 2020; Salamandane et al., 2020; Siwila et al., 2020). This study showed the occurrence of these two important human pathogenic species and their genotypes among raw and fresh horticultural products in different markets of different typologies in Maputo city. Using nested-PCR techniques, 2.8 and 1.3% of the samples monitored were positive for *G. duodenalis* and *E. bieneusi*, respectively. Based on the *bg* and ITS rRNA sequences analysis of *G. duodenalis* and *E. bieneusi*, respectively, four different *G. duodenalis* sequences (three novel and one known) all from assemblage B and three *E. bieneusi* genotypes (two novel and one known) from group 1 were characterized for the first time in fruits and vegetables from Maputo markets in Mozambique.

Much higher prevalence rates of *G. duodenalis* human infection are observed in developing countries in comparison to the developed regions (Dixon, 2021). The prevalence of *G. duodenalis* in Africa has been reported between rates from 0.3 to 62.2% according to the population studied (age, gender, symptoms, and immunological status) and the methodology used (Squire and Ryan, 2017; Damitie et al., 2018; Miambo et al., 2019; Yu et al., 2019; Belkessa et al., 2021; Messa et al., 2021). It is estimated that this protozoan causes about 28.2 million cases of diarrhea annually due to the ingestion of contaminated food (Ryan et al., 2019). *Giardia duodenalis* human infections are often associated with the consumption of contaminated raw fruits and vegetables, and the occurrence of the parasite cysts in these products has been reported in many countries with an average prevalence estimated of 4.8% (Li et al., 2020a). The 2.8% of *G. duodenalis* occurrence in the samples analyzed in our study is in accordance with the few available data from African countries that described 0–17.8% rates of this parasite in contaminated vegetables and fruits, reported in Morocco, Egypt, Libya, Ghana, Ethiopia, and Nigeria (Eraky et al., 2014; Istifanus and Panda, 2018; Kudah et al., 2018; Alemu et al., 2019; Berrouch et al., 2020; Li et al., 2020a; Sripanompong et al., 2021), mainly the leafy ones (Badri et al., 2022). Although the rate of incidence for *G. duodenalis* has been described as 0.11 in Africa (Hald et al., 2016), other studies mentioned the prevalence of *G. duodenalis* higher in developing countries (0.9–40.7%) when compared with developed countries (0.4–7.0%; Ryan and Cacciò, 2013; Wu et al., 2020b; Mozer et al., 2022).

As observed for *G. duodenalis*, the potential zoonotic pathogen *E. bieneusi* is described as a cause of symptomatic and asymptomatic infections in humans and other animals worldwide. There is limited information on *E. bieneusi* occurrence and genetic characterization in African countries, but the reported frequency rates of infection can range from 0.5 to 76.9%, mostly among HIV-infected patients, by PCR techniques (Muadica et al., 2020). More recently, Muadica et al. detected 0.7% (9/1,247) of *E. bieneusi*-positive samples in symptomatic and asymptomatic children in Mozambique (Muadica et al., 2020). Most human infections with *E. bieneusi* are thought to result from fecal-oral transmission of infectious spores through ingestion of contaminated food or water (Qiu et al., 2019). For instance, *E. bieneusi* has been identified in milk, raspberries, beans, and lettuce (Lee, 2008; Decraene et al., 2012), and the first foodborne outbreak caused by this species was reported in Sweden in 2009 (Decraene et al., 2012). More recently, a study mentioned a foodborne outbreak of *E. bieneusi* in Denmark (Michlmayr et al., 2022). Thus, foodborne transmission of *E. bieneusi* has been documented, and the contamination of vegetables and fruits with this pathogen was reported in China (PCR), Costa Rica (Ziehl-Nielsen stain), and Poland (staining or with fluorescence *in situ* hybridization). The average prevalence of the reported contamination among these studies was estimated as 3.6% (Li et al., 2020a). A lower frequency of contamination by *E. bieneusi* (1.3%) was detected in the vegetables and fruits analyzed in this study. Although this low rate may be associated with the difficulty in recovering these tiny microsporidia spores from the horticultural products analyzed, limiting their molecular detection, this finding may also constitute a positive indicator of safer products commercialized in these markets compared to others from different regions.

The presence of *G. duodenalis* was observed in fresh vegetables purchased in four municipal markets and *E. bieneusi* in fresh vegetables purchased in three markets and one supermarket. The higher occurrence of these pathogenic species in the main markets (open-air markets with limited or even absent proper sanitary conditions) than in the supermarkets monitored was expected and is certainly associated with their specific structural and environmental characteristics, which contribute to the contamination of food products in those former markets. However, in the other markets of different typologies surveyed, namely, in Central da Baixa market (a covered, well-structured market) and especially in the supermarket that have all the guaranteed resources, availability of water, market stalls and toilets, transport, handling and display of products for their safety, the produce contamination by one or both pathogenic species observed were not expected. The vegetables and fruits handled during preparation for display in the supermarket or even by the consumer can be one of the factors for the contamination that we observed (Salamandane et al., 2020, 2021b).

Both pathogens were mostly found in leafy vegetables (lettuce, point white cabbage, and parsley). Usually, high contamination rates of lettuce and other types of leafy vegetables (cabbage, parsley, and coriander) are also reported by other authors due to the uneven external surface of their leaves, which enables the easy entry and adherence of the *G. duodenalis* and *E. bieneusi* resistant forms (Rodrigues et al., 2020; Salamandane et al., 2020, 2021b; Badri et al., 2022).

In reports of contaminated vegetables and fruit worldwide, among the eight genetically distinct *G. duodenalis* assemblages (A–H) described to date, the zoonotic assemblages A and B were commonly detected (Li et al., 2020a). A few *Giardia* genotyping studies have been carried out in Africa; however, available reports show that five *G. duodenalis* assemblages (A, B, C, E, and F) have been identified in humans (Cai et al., 2021). In African countries, assemblage B was the most frequent among genotyped samples (19.5–100%) in 18 of 28 human population studies with assemblage A dominant (1.4–100%) in the remaining 10 reports (Cai et al., 2021). Recently, a study performed in Mozambique (Messa et al., 2021), in children aged under 5 years, reported a very low prevalence for assemblage A (10%) when compared with assemblage B (90%), following the normal tendency in sub-Saharan Africa. β*-*giardin sequence analysis from all nine *G. duodenalis* sequences identified in this study belong exclusively to assemblage B. Within the assemblage B sequences, four distinct *bg* sequences were formed: *bg* one sequence is an identical *bg* sequence to one previously reported; and the other three (BgMZ1, BgMZ2, and BgMZ3) display 99% of similarity to other *bg* sequences deposited in the reference database. The four different *bg* sequences identified were very similar with a mild difference between them and were relative to the reference sequences only from one to three single-nucleotide polymorphisms (SNP). Phylogenetic analysis of all nine *G. duodenalis* isolates, from the vegetables and fruits included in this study, indicates that all of them clustered closer to other sequences (Figure 1) previously detected in humans, other animals, and water, corroborating their potential role as a source of human infection and environmental contamination. In this study, the observed heterogeneity of assemblage B among *G. duodenalis bg* sequences was somewhat expected. This assemblage typically exhibits more genetic variability than assemblage A, as reported in the literature (Cai et al., 2021).

Genotyping tools and the phylogenetic analysis of *E. bieneusi* genotypes inferred by the genetic diversity of their ITS rRNA region recognized 11 genetic groups (Groups 1–11), figuring out their host specificity and zoonotic potential. Molecular studies on *E. bieneusi* worldwide stated that the occurrence of genotypes from Groups 1 (D, EbpC, and Type IV) and 2 (BEB4, BEB6, I, and J) are the most commonly identified (Li et al., 2019b, 2020b; Rahimi et al., 2021), where pigs and cattle are the main zoonotic reservoir of *E. bieneusi* (Li et al., 2019b; Zang et al., 2021). Despite the few studies on *E. bieneusi* in vegetables and water, some authors reported that D, BEB6, and E genotypes are the most frequent and with high zoonotic potential. In fact, the D genotype was identified also in chicken, water, humans, and vegetables (Javanmard et al., 2018; Cao et al., 2020; Rahimi et al., 2021). In Africa, molecular characterization remains rare and connected with livestock or clinical environments, performed only in a few countries such as Algeria, Ethiopia, Nigeria, South Africa, and recently in Mozambique (Akinbo et al., 2012; Samra et al., 2012; Laatamna et al., 2015; Stentiford et al., 2019; Muadica et al., 2020; Wegayehu et al., 2020). In another study working with stool specimens (Muadica et al., 2020), some known genotypes (Peru 11, Type IV and S2) were identified and described a new one HhMzEb1 (from Group 1) in Mozambique. None of the studies was associated with food or water or horticultural products. Nucleotide sequence analysis of the ITS region revealed a high degree of genetic diversity with a total of three distinct genotypes including one known (KIN-1) and two novel (EbMZ4 and EbMZ5) genotypes in the four *E. bieneusi* sequences obtained from the horticultural products monitored in this study. Phylogenetic analysis showed that both the known and the novel genotypes clustered within the previously designated zoonotic Group 1 include genotypes with low host specificity and the potential for zoonotic and cross-species transmission (Figure 2). All the genotypes identified in this study clustered very closer with other genotypes (e.g., D, Ebp C; Figure 2) that display a high zoonotic transmission potential (Li et al., 2020b).

The contamination of fresh horticultural products with the infectious stages of parasites may occur at any of the distinct points from the farm level to the food handler/consumer level. At the farm level, the contamination of fresh vegetables and fruits may occur during production, harvesting, packaging, transport, or selling. Contamination with *Giardia* cysts and *E. bieneusi* spores can occur directly from the hands of infected farm workers (or their equipment) or those who are in close contact with infected individuals; through the use of animal or human feces as manure for farmland; or direct access to infected cattle and other animals to crops. Indirect contamination of horticultural products at the production zone can occur by using irrigation water contaminated with feces; mixing pesticides; or washing horticultural products, hands, or equipment (Dixon, 2021). The direct contamination of fresh produce by infected food handlers, or who have been in close contact with infected persons, is probably a main contributor and has been identified as the direct cause of a number of outbreaks (Dixon, 2021). Although, in this study, *G. duodenalis* and *E. bieneusi* were not detected in the production zone (Infulene Valley) by the molecular techniques, it does not mean for sure that the products were not contaminated at this point. For instance, *G. duodenalis*, like other intestinal parasites, were detected in this zone by the authors (Salamandane et al., 2021b), in a previous study with microscopic methods. In this study, different *G. duodenalis bg* sequences from assemblage B, as well as similar *E. bieneusi* genotypes or *G. duodenalis* assemblage B, were detected in the same market/supermarket. Also, similar *E. bieneusi* genotypes/*G. duodenalis bg* sequences from assemblage B were identified in distinct markets (Table 1). All this reflection leads us to think that the possible contamination of horticultural products in markets and supermarkets in our study may have a similar origin, which is in agreement with other authors who report that post-harvest contamination can occur mainly during packaging, transport, and handling of the products (Vizon et al., 2019; Salamandane et al., 2021a).

The recognition of these intestinal pathogens in fresh vegetables and fruits not only allows this study to be framed within the strict scope of zoonotic diseases, helping to clarify the epidemiology of *G. duodenalis* and *E. bieneusi* in the country but also opens doors to other dimensions. There is a particular emphasis on the environmental dimension and its role in global homeostasis, as advocated in the context of the One Health approach. In addition to alerting to the risk for human consumption of these products, directly improving health population outcomes, this study suggests the existence of contamination at an environmental level. Such contamination may occur through animals and wastewater polluted with human and animal waste during various processes of production, transport, and sale of these products. Therefore, our findings indirectly reinforce the need to develop better epidemiological surveillance systems for enteric pathogens in the area and to adopt measures such as improving sanitary conditions and ensuring safe water and food to prevent and control these diseases. These measures will bring benefits to the health of people, animals, and ecosystems.

## 5. Strengths and limitations of the study

Our research, to the best of our knowledge, represents a groundbreaking study on the detection of *G. duodenalis* and *E. bieneusi*, as well as the assessment of their genetic heterogeneity. We isolated these organisms from raw horticultural products obtained from eight different types of markets in Maputo, Mozambique. Additionally, this study included a significant number of horticultural products with two different food matrices (leafy vegetables and fruits) that were randomly purchased in triplicate from the most popular and frequently visited markets in Maputo city, during the two main seasons of the year. By employing molecular tools, we not only demonstrated the presence of potential zoonotic assemblages/genotypes of *G. duodenalis* and *E. bieneusi* among the fresh fruits and vegetables examined but also highlighted the usefulness of these tools in tracing the sources of food contamination.

However, our study has certain limitations associated with the methodology we adopted, particularly the protocols used for recovering cysts/spores from horticultural product extracts and the molecular tools employed. These limitations are primarily due to the unavailability of certain technical resources, such as a fluorescent microscope, and the constraints of working in the field. The only standardized method currently available for detecting intestinal protist parasites in food matrices is ISO 18744:2016, which was developed and optimized for the detection and enumeration of *Cryptosporidium* and *Giardia* in fresh leafy green vegetables and berry fruits. However, this method is neither suitable for routine analysis or monitoring in a hazard analysis plan and critical control points (Chalmers et al., 2020) nor does it enable the determination of species or genotypes (Efstratiou et al., 2017b). Therefore, to overcome this limitation, we reviewed several protocols described in the literature for detaching and concentrating the transmissive stages of intestinal pathogens from vegetable matrices. Then, we selected the most widely accepted approach, considering the specific size and food matrices of the products studied and the conditions under which the fieldwork was conducted. Additionally, it is important to mention that the concentration and purification processes for the main protist transmissive stages recommend conducting initial spiking experiments with known amounts of (oo)cysts/spores to evaluate the median percentage recovery of the chosen method. However, this study was unable to perform such a procedure, and as a result, the recovery rates of these methodologies were not estimated. This strongly suggests that the reported PCR-positive rate here is likely an underestimate of the true rate. Furthermore, due to limitations in resources and technical conditions, it was not possible to perform a multilocus sequence genotyping of *G. duodenalis*. If a multi-copy marker, such as the SSU rRNA gene, had been selected, it would have increased the sensitivity of detecting parasite cysts in vegetable product extracts. Instead, we utilized the *bg* locus, which is a single-copy gene but strikes a balance between very specific detection and genetic discrimination. While these methodology choices were made based on the available resources and technical conditions in the field, certainly, they may have led to an underestimation of both the positivity rate for *Giardia* and *E. bieneusi* reported in this study.

Even though it was not within the scope of this study, the absence of available technical conditions represents another limitation. Specifically, we were unable to conduct viability and infectivity assays on the transmissive stages of the detected protists in the horticultural products. Consequently, by solely focusing on the detection and genotyping of these microorganisms without assessing their viability and infectivity, the true impact of these agents on public health may have been underestimated. However, it is important to note that the transmissive stages of these important pathogens were found in raw products that had not undergone any cooking or freezing processes prior to ingestion by humans or other animals. Considering the high resistance of cysts and spores to adverse environmental conditions, as well as the endemic nature of both microorganisms in the country, these findings undoubtedly suggest concerns regarding public health and warrant further investigation in future studies.

## 6. Conclusion

To the best of our knowledge, this study presents the first data on the detection and genetic characterization of the pathogenic *G. duodenalis* and *E. bieneusi* in horticultural products collected in dry and rainy seasons from different typology markets in Maputo city, Mozambique. In addition, this study suggests the presence of potential zoonotic assemblages or genotypes of these two important pathogens in vegetables and fruits, commercialized in the markets most used by the population of the city and therefore can be a source of human infection by gastrointestinal parasitic pathogens. Another major concern is the fact that many people tend to consume these horticultural products raw, in salads, and other typical street food available in markets and/or nearby. Although no viability or infectivity assays were conducted to assess the actual impact of these protists on public health, this study suggests a risk associated with consuming raw horticultural products contaminated with these pathogenic intestinal species. It emphasizes the importance of implementing improved safety practices throughout the production, transport, and handling of these products. These measures should be adopted by producers, vendors, and consumers to minimize the potential for foodborne outbreaks.

## Data availability statement

The original contributions presented in the study are included in the article/supplementary material, further inquiries can be directed to the corresponding authors.

## Author contributions

ML and OM: conceptualization, project administration, and supervision. CS, ML, SA, and OM: investigation. CS, SA, LX, and ML: methodology. CS, ML, and LX: software. CS and ML: writing—original draft. LX and OM: writing—review and editing. All authors have read and agreed to the published version of the manuscript.

## References

[B1] Ahmed S. A. Guerrero Flórez M. Karanis P. Efstratiou A. Ongerth J. Karanis P. (2018). The impact of water crises and climate changes on the transmission of protozoan parasites in Africa. Water Res. 112, 96–112. 10.1080/20477724.2018.1523778 30332341 PMC6381522

[B2] Akinbo F. O. Okaka C. E. Omoregie R. Dearen T. Leon E. T. Xiao L. (2012). Molecular epidemiologic characterization of *Enterocytozoon bieneusi* in HIV-infected persons in Benin City, Nigeria. Am. J. Trop. Med. Hyg. 86, 441–445. 10.4269/ajtmh.2012.11-0548 22403314 PMC3284359

[B3] Alemu G. Mama M. Misker D. Haftu D. (2019). Parasitic contamination of vegetables marketed in Arba Minch town, southern Ethiopia. BMC Infect. Dis. 19. 10.1186/s12879-019-4020-5 31088390 PMC6515664

[B4] Archer J. O'Halloran L. Al-Shehri H. Summers S. Bhattacharyya T. Kabaterine N. B.. (2020). Intestinal *Schistosomiasis* and *Giardiasis* co-infection in Sub-Saharan Africa: can a One Health approach improve control of each waterborne parasite simultaneously? Trop. Med. Infect. Dis. 5, 137. 10.3390/tropicalmed5030137 32854435 PMC7558413

[B5] Badri M. Olfatifar M. Karim M. R. Modirian E. Houshmand E. Abdoli A.. (2022). Global prevalence of intestinal protozoan contamination in vegetables and fruits: a systematic review and meta-analysis. Food Control 133, 108656. 10.1016/j.foodcont.2021.108656

[B6] Bahramian B. Afshari A. Kiani B. Sani M. A. Hashemi M. (2021). The prevalence of foodborne parasites in raw vegetables in Iran: a comprehensive systematic review and meta-analysis. J. Environ. Heal. Sci. Eng. 19, 2027–2045. 10.1007/s40201-021-00714-w 34900321 PMC8617144

[B7] Baldursson S. Karanis P. (2011). Waterborne transmission of protozoan parasites: review of worldwide outbreaks—An update 2004-2010. Water Res. 45, 6603–6614. 10.1016/j.watres.2011.10.013 22048017

[B8] Belkessa S. Thomas-Lopez D. Houali K. Ghalmi F. Stensvold C. R. (2021). Molecular characterization of giardia duodenalis in children and adults sampled in algeria. Microorganisms 9, 1–11. 10.3390/microorganisms9010054 33379186 PMC7823855

[B9] Berrouch S. Escotte-Binet S. Harrak R. Huguenin A. Flori P. Favennec L.. (2020). Detection methods and prevalence of transmission stages of *Toxoplasma gondii, Giardia duodenalis* and *Cryptosporidium* spp. in fresh vegetables: a review. Parasitology 147, 516–532. 10.1017/S0031182020000086 31965956 PMC10317687

[B10] Cacciò S. M. De Giacomo M. Pozio E. (2002). Sequence analysis of the β-giardin gene and development of a polymerase chain reaction-restriction fragment length polymorphism assay to genotype *Giardia duodenalis* cysts from human faecal samples. Int. J. Parasitol. 32, 1023–1030. 10.1016/S0020-7519(02)00068-1 12076631

[B11] Cacciò S. M. Lalle M. Svärd S. G. (2018). Host specificity in the *Giardia duodenalis* species complex. Infect. Genet. Evol. 66, 335–345. 10.1016/j.meegid.2017.12.001 29225147

[B12] Cai W. Ryan U. Xiao L. Feng Y. (2021). Zoonotic giardiasis: an update. Parasitol. Res. 120, 4199–4218. 10.1007/s00436-021-07325-2 34623485

[B13] Cao S. Xu M. Jiang Y. Liu H. Yuan Z. Sun L.. (2020). Prevalence and genetic characterization of *Cryptosporidium, Giardia* and *Enterocytozoon* in chickens from Ezhou, Hubei, China. Front. Vet. Sci. 7, 30. 10.3389/fvets.2020.00030 32083107 PMC7005591

[B14] Cao Y. Tong Q. Zhao C. Maimaiti A. Chuai L. Wang J.. (2021). Molecular detection and genotyping of *Enterocytozoon bieneusi* in pet dogs in Xinjiang, Northwestern China. Parasite 28, 2021057. 10.1051/parasite/2021057 34283021 PMC8290926

[B15] Chalmers R. M. Robertson L. J. Dorny P. Jordan S. Kärssin A. Katzer F.. (2020). Parasite detection in food: current status and future needs for validation. Trends Food Sci. Technol. 99, 337–350. 10.1016/j.tifs.2020.03.01135146143

[B16] Chen L. Zhao J. Li N. Guo Y. Feng Y. Feng Y.. (2019). Genotypes and public health potential of Enterocytozoon bieneusi and Giardia duodenalis in crab-eating macaques. Parasit. Vect. 12, 1–11. 10.1186/s13071-019-3511-y 31118092 PMC6530032

[B17] Cook N. Nichols R. A. B. Wilkinson N. Paton C. A. Barker K. Smith H. V. (2007). Development of a method for detection of *Giardia duodenalis* cysts on lettuce and for simultaneous analysis of salad products for the presence of *Giardia* cysts and *Cryptosporidium oocysts*. Appl. Environ. Microbiol. 73, 7388–7391. 10.1128/AEM.00552-07 17890337 PMC2168210

[B18] Cunha M. J. R. Cury M. C. Santín M. (2017). Molecular identification of *Enterocytozoon bieneusi, Cryptosporidium*, and *Giardia* in Brazilian captive birds. Parasitol. Res. 116, 487–493. 10.1007/s00436-016-5309-6 27815734

[B19] Dalu T. Barson M. Nhiwatiwa T. (2011). Impact of intestinal microorganisms and protozoan parasites on drinking water quality in Harare, Zimbabwe. J. Water Sanit. Hyg. Dev. 1, 153–163. 10.2166/washdev.2011.049

[B20] Damitie M. Mekonnen Z. Getahun T. Santiago D. Leyns L. (2018). Molecular epidemiology of *Giardia duodenalis* infection in humans in Southern Ethiopia: a triosephosphate isomerase gene-targeted analysis. Infect. Dis. Poverty 7, 1–10. 10.1186/s40249-018-0397-4 29502512 PMC5836388

[B21] Decraene V. Lebbad M. Botero-Kleiven S. Gustavsson A. M. Löfdahl M. (2012). First reported foodborne outbreak associated with microsporidia, Sweden, October 2009. Epidemiol. Infect. 140, 519–527. 10.1017/S095026881100077X 21733266 PMC3267097

[B22] Dixon B. R. (2021). Giardia duodenalis in humans and animals—Transmission and disease. Res. Vet. Sci. 135, 283–289. 10.1016/j.rvsc.2020.09.034 33066992

[B23] Efstratiou A. Ongerth J. Karanis P. (2017b). Evolution of monitoring for *Giardia* and *Cryptosporidium* in water. Water Res. 123, 96–112. 10.1016/j.watres.2017.06.042 28651085

[B24] Efstratiou A. Ongerth J. E. Karanis P. (2017a). Waterborne transmission of protozoan parasites: review of worldwide outbreaks—An update 2011–2016. Water Res. 114, 36. 10.1016/j.watres.2017.01.036 28214721

[B25] Eraky M. A. Rashed S. M. Nasr M. E. S. El-Hamshary A. M. S. Salah El-Ghannam A. (2014). Parasitic contamination of commonly consumed fresh leafy vegetables in Benha, Egypt. J. Parasitol. Res. 2014, 7. 10.1155/2014/613960 25024845 PMC4084512

[B26] Ferreira F. P. Caldart E. T. Freire R. L. Mitsuka-Breganó R. de Freitas F. M. Miura A. C.. (2018). The effect of water source and soil supplementation on parasite contamination in organic vegetable gardens. Rev. Bras. Parasitol. Vet. 27, 327–337. 10.1590/s1984-296120180050 30183998

[B27] Hald T. Aspinall W. Devleesschauwer B. Cooke R. Corrigan T. Havelaar A. H.. (2016). World Health Organization estimates of the relative contributions of food to the burden of disease due to selected foodborne hazards: a structured expert elicitation. PLoS ONE 11, 1–35. 10.1371/journal.pone.0145839 26784029 PMC4718673

[B28] Hassan E. M. Örmeci B. DeRosa M. C. Dixon B. R. Sattar S. A. Iqbal A. (2021). A review of *Cryptosporidium* spp. and their detection in water. Water Sci. Technol. 83, 1–25. 10.2166/wst.2020.515 33460403

[B29] Istifanus W. A. Panda S. M. (2018). Parasitic agents in fresh fruits and vegetables sold in open markets in Bauchi, Nigeria. J. Food Qual. Hazards Control 5, 84–88. 10.29252/jfqhc.5.3.84

[B30] Javanmard E. Mirjalali H. Niyyati M. Jalilzadeh E. Seyed Tabaei S. J. Asadzadeh Aghdaei H.. (2018). Molecular and phylogenetic evidences of dispersion of human-infecting microsporidia to vegetable farms via irrigation with treated wastewater: one-year follow up. Int. J. Hyg. Environ. Health 221, 642–651. 10.1016/j.ijheh.2018.03.007 29627259

[B31] Javanmard E. Mirsamadi E. S. Olfatifar M. Ghasemi E. Saki F. Mirjalali H.. (2020). Prevalence of *Cryptosporidium* and *Giardia* in vegetables in Iran: a nineteen-years meta-analysis review. J. Environ. Heal. Sci. Eng. 18, 1629–1641. 10.1007/s40201-020-00493-w 33312667 PMC7721826

[B32] Junaidi J. Cahyaningsih U. Purnawarman T. Latif H. Sudarnika E. Hayati Z.. (2020). “*Entamoeba histolytica* Neglected Tropical Diseases (NTDs) agents that infect humans and some other mammals: a review,” in E3S Web of Conferences (Banda Aceh). 10.1051/e3sconf/202015101019

[B33] Kudah C. Sovoe S. Baiden F. (2018). Parasitic contamination of commonly consumed vegetables in two markets in Ghana. Ghana Med. J. 52, 88–93. 10.4314/gmj.v52i2.5 30662081 PMC6326541

[B34] Laatamna A. E. Wagnerová P. Sak B. Květonová D. Xiao L. Rost M.. (2015). Microsporidia and *Cryptosporidium* in horses and donkeys in Algeria: detection of a novel *Cryptosporidium hominis* subtype family (Ik) in a horse. Vet. Parasitol. 208, 135–142. 10.1016/j.vetpar.2015.01.007 25638716

[B35] Lalle M. Pozio E. Capelli G. Bruschi F. Crotti D. Cacciò S. M. (2005). Genetic heterogeneity at the β-giardin locus among human and animal isolates of *Giardia duodenalis* and identification of potentially zoonotic subgenotypes. Int. J. Parasitol. 35, 207–213. 10.1016/j.ijpara.2004.10.022 15710441

[B36] Lee J. H. (2008). Molecular detection of *Enterocytozoon bieneusi* and identification of a potentially human-pathogenic genotype in milk. Appl. Environ. Microbiol. 74, 1664–1666. 10.1128/AEM.02110-07 18192409 PMC2258614

[B37] Li J. Shi K. Sun F. Li T. Wang R. Zhang S.. (2019a). Identification of human pathogenic *Enterocytozoon bieneusi, Cyclospora cayetanensis*, and *Cryptosporidium parvum* on the surfaces of vegetables and fruits in Henan, China. Int. J. Food Microbiol. 307, 108292. 10.1016/j.ijfoodmicro.2019.108292 31430663

[B38] Li J. Wang Z. Karim M. R. Zhang L. (2020a). Detection of human intestinal protozoan parasites in vegetables and fruits: a review. Parasit. Vect. 13, 380. 10.1186/s13071-020-04255-3 32727529 PMC7392835

[B39] Li N. Xiao L. Wang L. Zhao S. Zhao X. Duan L.. (2012). Molecular surveillance of *Cryptosporidium* spp., *Giardia duodenalis*, and *Enterocytozoon bieneusi* by genotyping and subtyping parasites in wastewater. PLoS Negl. Trop. Dis. 6, 1809. 10.1371/journal.pntd.0001809 22970334 PMC3435239

[B40] Li W. Feng Y. Santin M. (2019b). Host specificity of *Enterocytozoon bieneusiand* public health implications. Trends Parasitol. 35, 436–451. 10.1016/j.pt.2019.04.004 31076351

[B41] Li W. Feng Y. Xiao L. (2020b). Diagnosis and molecular typing of *Enterocytozoon bieneusi*: the significant role of domestic animals in transmission of human microsporidiosis. Res. Vet. Sci. 133, 251–261. 10.1016/j.rvsc.2020.09.030 33035931

[B42] Lobo M. L. Augusto J. Antunes F. Ceita J. Xiao L. Codices V.. (2014). *Cryptosporidium* spp., *Giardia duodenalis, Enterocytozoon bieneusi* and Other intestinal parasites in young children in Lobata Province, Democratic Republic of São Tomé and Principe. PLoS ONE 9, e97708. 10.1371/journal.pone.0097708 24846205 PMC4028242

[B43] Lobo M. L. Xiao L. Antunes F. Matos O. (2012). Microsporidia as emerging pathogens and the implication for public health: a 10-year study on HIV-positive and -negative patients. Int. J. Parasitol. 42, 197–205. 10.1016/j.ijpara.2011.12.002 22265899

[B44] Matos O. Lobo M. L. Xiao L. (2012). Epidemiology of *Enterocytozoon bieneusi* infection in humans. J. Parasitol. Res. 2012, 981424. 10.1155/2012/981424 23091702 PMC3469256

[B45] Messa A. Köster P. C. Garrine M. Gilchrist C. Bartelt L. A. Nhampossa T.. (2021). Molecular diversity of *Giardia duodenalis* in children under 5 years from the manhiça district, southern mozambique enrolled in a matched case-control study on the aetiology of diarrhoea. PLoS Negl. Trop. Dis. 15, 1–24. 10.1371/journal.pntd.0008987 33465074 PMC7846004

[B46] Miambo R. D. Laitela B. Malatji M. P. Afonso S. M. S. Junior A. P. Lindh J.. (2019). Prevalence of *Giardia* and *Cryptosporidium* in young livestock and dogs in magude district of Maputo Province, Mozambique. Onderstepoort J. Vet. Res. 86, a1709. 10.4102/ojvr.v86i1.1709 31478737 PMC6739555

[B47] Michlmayr D. Alves de Sousa L. Müller L. Jokelainen P. Ethelberg S. Vestergaard L. S.. (2022). Incubation period, spore shedding duration, and symptoms of *Enterocytozoon bieneusi* genotype C infection in a foodborne outbreak in Denmark, 2020. Clin. Infect. Dis. 75, 468–475. 10.1093/cid/ciab949 34791090 PMC9427152

[B48] Monge R. Chinchilla M. (1996). Presence of *Cryptosporidium oocysts* in fresh vegetables. J. Food Prot. 59, 202–203. 10.4315/0362-028X-59.2.202 31158995

[B49] Mozer S. Abdulwahhab I. G. Azaawie A. L. A. F. (2022). Extraction of the DNA of *Giardia lamblia* isolated from vegetables and fruits in a simplified way and its diagnosis using Nested-PCR. J. Parasit. Dis. 46, 771–775. 10.1007/s12639-022-01484-4 36091271 PMC9458841

[B50] Mtapuri-Zinyowera S. Ruhanya V. Midzi N. Berejena C. Chin'Ombe N. Nziramasanga P.. (2014). Human parasitic protozoa in drinking water sources in rural Zimbabwe and their link to HIV infection. Germs 4, 86–91. 10.11599/germs.2014.1061 25505741 PMC4258399

[B51] Muadica A. S. Messa A. Dashti A. Balasegaram S. Santin M. Manjate F.. (2020). First identification of genotypes of *Enterocytozoon bieneusi* (Microsporidia) among symptomatic and asymptomatic children in mozambique. PLoS Negl. Trop. Dis. 14, 1–19. 10.1371/journal.pntd.0008419 32603325 PMC7357779

[B52] Qiu L. Xia W. Li W. Ping J. Ding S. Liu H. (2019). The prevalence of microsporidia in China: a systematic review and meta-analysis opeN. Sci. Rep. 9, 3174. 10.1038/s41598-019-39290-3 30816168 PMC6395699

[B53] Rafael K. Marchioro A. A. Colli C. M. Tiyo B. T. Evangelista F. F. Bezagio R. C.. (2017). Genotyping of *Giardia duodenalis* in vegetables cultivated with organic and chemical fertilizer from street markets and community vegetable gardens in a region of Southern Brazil. Trans. R. Soc. Trop. Med. Hyg. 111, 540–545. 10.1093/trstmh/try013 29518239

[B54] Rafiei A. Baghlaninezhad R. Köster P. C. Bailo B. de Mingo M. H. Carmena D.. (2020). Multilocus genotyping of *Giardia duodenalis* in Southwestern Iran. A community survey. PLoS ONE 15, 228317. 10.1371/journal.pone.0228317 32027684 PMC7004373

[B55] Rahimi H. M. Mirjalali H. Zali M. R. (2021). Molecular epidemiology and genotype/subtype distribution of *Blastocystis* sp., *Enterocytozoon bieneusi*, and *Encephalitozoon* spp. in livestock: concern for emerging zoonotic infections. Sci. Rep. 11, 17467. 10.1038/s41598-021-96960-x 34471179 PMC8410837

[B56] Rodrigues A. C. da Silva M. D. C. Pereira R. Â. S. Pinto L. C. (2020). Prevalence of contamination by intestinal parasites in vegetables (*Lactuca sativa* L. and *Coriandrum sativum* L.) sold in markets in Belém, northern Brazil. J. Sci. Food Agric. 100, 2859–2865. 10.1002/jsfa.10265 31953861

[B57] Ryan U. Cacciò S. M. (2013). Zoonotic potential of *Giardia*. Int. J. Parasitol. 43, 943–956. 10.1016/j.ijpara.2013.06.001 23856595

[B58] Ryan U. Hijjawi N. Feng Y. Xiao L. (2019). *Giardia*: an under-reported foodborne parasite. Int. J. Parasitol. 49, 1–11. 10.1016/j.ijpara.2018.07.003 30391227

[B59] Salamandane A. Silva A. C. Brito L. Malfeito-Ferreira M. (2021a). Microbiological assessment of street foods at the point of sale in Maputo (Mozambique). Food Qual. Saf . 5, 1–9. 10.1093/fqsafe/fyaa030

[B60] Salamandane C. Fonseca F. Afonso S. Lobo M. L. Antunes F. Matos O. (2020). Handling of fresh vegetables: knowledge, hygienic behavior of vendors, public health in maputo markets, Mozambique. Int. J. Environ. Res. Public Health 17, 6302. 10.3390/ijerph17176302 32872524 PMC7504209

[B61] Salamandane C. Lobo M. L. Afonso S. Miambo R. Matos O. (2021b). Occurrence of intestinal parasites of public health significance in fresh horticultural products sold in Maputo markets and supermarkets, Mozambique. Microorganisms 9, 1806. 10.3390/microorganisms9091806 34576702 PMC8469142

[B62] Samra N. A. Thompson P. N. Jori F. Zhang H. Xiao L. (2012). *Enterocytozoon bieneusi* at the wildlife/livestock interface of the Kruger National Park, South Africa. Vet. Parasitol. 190, 587–590. 10.1016/j.vetpar.2012.06.031 22824060

[B63] Santin M. Fayer R. (2009). *Enterocytozoon bieneusi* genotype nomenclature based on the internal transcribed spacer sequence: a consensus. J. Eukaryot. Microbiol. 56, 34–38. 10.1111/j.1550-7408.2008.00380.x 19335772

[B64] Siwila J. Mwaba F. Chidumayo N. Mubanga C. (2020). Food and waterborne protozoan parasites: the African perspective. Food Waterborne Parasitol. 20, e00088. 10.1016/j.fawpar.2020.e00088 32995582 PMC7502820

[B65] Squire S. A. Ryan U. (2017). *Cryptosporidium* and *Giardia* in Africa: current and future challenges. Parasit. Vect. 10, 1–32. 10.1186/s13071-017-2111-y 28427454 PMC5397716

[B66] Sripanompong J. Tipayamongkholgul M. Mahittikron A. Kosaisavee V. (2021). *Giardia duodenalis* contamination of fresh vegetables in a wholesale market. Thai J. Public Heal. 51, 276–283.

[B67] Stentiford G. D. Bass D. Williams B. P. (2019). Ultimate opportunists—the emergent enterocytozoon group microsporidia. PLoS Pathog. 15, e1007668. 10.1371/journal.ppat.1007668 31048922 PMC6497299

[B68] Stentiford G. D. Becnel J. J. Weiss L. M. Keeling P. J. Didier E. S. Williams B. A. P. P.. (2016). Microsporidia—Emergent pathogens in the global food chain. Trends Parasitol. 32, 336–348. 10.1016/j.pt.2015.12.004 26796229 PMC4818719

[B69] Thompson R. C. A. Palmer C. S. O'Handley R. (2008). The public health and clinical significance of *Giardia* and *Cryptosporidium* in domestic animals. Vet. J. 177, 18–25. 10.1016/j.tvjl.2007.09.022 18032076 PMC7128580

[B70] Utaaker K. Kumar A. Joshi H. Chaudhary S. Robertson L. (2017). Checking the detail in retail: occurrence of *Cryptosporidium* and *Giardia* on vegetables sold across different counters in Chandigarh, India. Int. J. Food Microbiol. 263, 1–8. 10.1016/j.ijfoodmicro.2017.09.020 28988154

[B71] Vizon K. C. C. Battad Z. G. Castillo D. S. C. (2019). Contamination of food-borne parasites from green-leafy vegetables sold in public markets of San Jose City, Nueva Ecija, Philippines. J. Parasit. Dis. 43, 651–657. 10.1007/s12639-019-01144-0 31749537 PMC6841888

[B72] Wang X. T. Wang R. J. Ren G. J. Yu Z. Q. Zhang L. X. Zhang S. Y.. (2016). Multilocus genotyping of *Giardia duodenalis* and *Enterocytozoon bieneusi* in dairy and native beef (Qinchuan) calves in Shaanxi province, northwestern China. Parasitol. Res. 115, 1355–1361. 10.1007/s00436-016-4908-6 26782809

[B73] Wegayehu T. Li J. Karim M. R. Zhang L. (2020). Molecular characterization and phylogenetic analysis of *Enterocytozoon bieneusi* in lambs in Oromia Special Zone, Central Ethiopia. Front. Vet. Sci. 7, 6. 10.3389/fvets.2020.00006 32083097 PMC7001644

[B74] Wu Y. Chen Y. Chang Y. Zhang X. Li D. Wang L.. (2020a). Genotyping and identification of *Cryptosporidium* spp., *Giardia duodenalis* and *Enterocytozoon bieneusi* from free–range Tibetan yellow cattle and cattle–yak in Tibet, China. Acta Trop. 212, 105671. 10.1016/j.actatropica.2020.105671 32822671

[B75] Wu Y. Gong B. Liu X. Jiang Y. Cao J. Yao L.. (2020b). Identification of uncommon *Cryptosporidium* viatorum (a novel subtype XVcA2G1c) and *Cryptosporidium andersoni* as well as common *Giardia duodenalis* assemblages A and B in humans in Myanmar. Front. Cell. Infect. Microbiol. 10, 614053. 10.3389/fcimb.2020.614053 33324584 PMC7724083

[B76] Xiao L. Feng Y. (2017). Molecular epidemiologic tools for waterborne pathogens *Cryptosporidium* spp. and *Giardia duodenalis*. Food Waterborne Parasitol. 2, 8–9. 10.1016/j.fawpar.2017.09.002 32095639 PMC7034008

[B77] Yaoyu F. Xiao L. (2011). Zoonotic potential and molecular epidemiology of *Giardia* species and giardiasis. Clin. Microbiol. Rev. 24, 110–140. 10.1128/CMR.00033-10 21233509 PMC3021202

[B78] Yu F. Amer S. Qi M. Wang R. Wang Y. Zhang S.. (2019). Multilocus genotyping of *Giardia duodenalis* isolated from patients in Egypt. Acta Trop. 196, 66–71. 10.1016/j.actatropica.2019.05.012 31100269

[B79] Zang M. Li J. Tang C. Ding S. Huang W. Qin Q.. (2021). Prevalence and phylogenetic analysis of microsporidium *Enterocytozoon bieneusi* in diarrheal patients. Pathogens 10, 1–10. 10.3390/pathogens10020128 33513788 PMC7912502

[B80] Zhang Y. Mi R. Yang J. Wang J. Gong H. Huang Y.. (2020). *Enterocytozoon bieneusi* genotypes in farmed goats and sheep in Ningxia, China. Infect. Genet. Evol. 85, 104559. 10.1016/j.meegid.2020.104559 32961363

