# Corrigendum: Exploring genetic variability of *Giardia duodenalis* and *Enterocytozoon bieneusi* in raw vegetables and fruits: implications for food safety and public health in Mozambique

**DOI:** 10.3389/fmicb.2023.1275235

**Published:** 2023-08-28

**Authors:** Cátia Salamandane, Maria Luísa Lobo, Sónia Afonso, Lihua Xiao, Olga Matos

**Affiliations:** ^1^Group of Opportunistic Protozoa/HIV and Other Protozoa, Global Health and Tropical Medicine, Medical Parasitology Unit, Instituto de Higiene e Medicina Tropical, Universidade Nova de Lisboa, Lisboa, Portugal; ^2^Nova School of Business and Economics, Universidade Nova de Lisboa, Carcavelos, Portugal; ^3^Faculdade de Ciências de Saúde, Universidade Lúrio, Nampula, Mozambique; ^4^Parasitology Department of Veterinary Faculty, Universidade Eduardo Mondlane, Maputo, Mozambique; ^5^College of Veterinary Medicine, South China Agricultural University, Guangzhou, Guangdong, China; ^6^Environmental Health Institute, Faculdade de Medicina da Universidade de Lisboa, Lisboa, Portugal

**Keywords:** intestinal protozoa, microsporidia, raw horticultural products, foodborne diseases, zoonotic transmission, Maputo, One Health

In the published article, there was an error in the **Funding statement**. An incorrect grant number was inserted and a funding source was omitted. The correct funding statement appears below:

This research was funded by Fundação para a Ciência e a Tecnologia (FCT) with a scholarship reference SFRH/BD/135355/2017 from TropikMan Doctoral Program, Lisboa, Portugal and the funds to GHTM – UID/04413/2020 and LA – REAL – LA/P/0117/2020.

The authors apologize for this error and state that this does not change the scientific conclusions of the article in any way. The original article has been updated.

